# AI recommendation vs. crowdsourced recommendation vs. travel expert recommendation: The moderating role of consumption goal on travel destination decision

**DOI:** 10.1371/journal.pone.0318719

**Published:** 2025-03-19

**Authors:** Young Eun Park, Hyunsang Son

**Affiliations:** 1 Department of Public Relations and Advertising, Sookmyung Women’s University, Seoul, South Korea; 2 Department of Marketing, Information, and Decision Sciences, Anderson School of Management, University of New Mexico, Albuquerque, New Mexico, United States of America; The Hong Kong Polytechnic University, HONG KONG

## Abstract

This research investigates the effects of recommender type (AI versus crowdsourcing versus travel expert) and consumption goal (utilitarian versus hedonic) on consumer responses to travel destination recommendation email advertisements. Across two experiments, findings reveal that consumers held the most favorable attitude toward email advertisements featuring travel expert recommendations compared to crowdsourced or AI-generated suggestions. Notably, this study highlights the moderating role of consumption goal type (utilitarian vs. hedonic) in shaping attitudes toward the recommendation source. Specifically, consumers preferred ads featuring AI-generated recommendations for utilitarian travel goals (e.g., business trips), while for hedonic travel goals (e.g., romantic getaways), they favored ads with crowdsourced suggestions. Through serial mediation (Study 1) and moderated serial mediation (Study 2), this research further elucidates the underlying psychological mechanisms driving these effects.

## Introduction

In 2023, international tourism ended at 88% of pre-pandemic levels, with more than 1.3 billion travelers worldwide, and it is expected to fully recover at a pre-pandemic level in 2024 [1]. The tourism industry contributed 7.6% of global GDP (7.7 trillion USD) and created 22 million new jobs in 2022 [2]. Marketers and advertisers have recognized the travel industry’s impact on the economy and have investigated a significant amount of resources for successful marketing campaigns, such as developing effective advertising strategies [3–6], building a successful loyalty program [7,8], and encouraging tourists to participate in sustainable tourism programs [9].

One important trend in the tourism industry is its rapid adoption of digital technology [10], including virtual reality [11,12], big data analysis [e.g., 13–15], and co-creation [16], to develop marketing strategies for the tourism industry. One notable integration of digital technology in the tourism industry is the adoption of co-creation [17] in travel destination selection. In 2018, Expedia, in collaboration with Hangzhou Tourism, suggested traveling to Hangzhou, China, and consumers were able to create their own trips by personalizing their preferences [18].

More recently, the emergence and adoption of artificial intelligence (AI) technology has brought about a dramatic revolution in the tourism industry. Indeed, it is already widely adopted. For example, many hotels, such as The Cosmopolitan Hotel in Las Vegas and Aloft Hotels, have introduced robotic butlers utilizing chatbots to respond to customers’ queries and demands more quickly [19]. Hilton also launched Connie, a Watson-enabled robot concierge, to help hotel guests find local attractions and dining places [20]. Perhaps the most promising area for utilizing AI in the travel industry is travel destination recommendation services. According to booking.com, 29% of travelers are comfortable letting a computer recommend their next trip based on their previous travel history, and 50% of customers mention that they do not care about the recommender of the travel destination as long as their questions are answered [21].

By recognizing emerging trends in the travel industry, this study investigates which travel destination recommendations yield the most favorable consumer evaluations. While AI-generated recommendations initially appeared to benefit both companies and consumers [22–24], many studies have highlighted the backfire effects of AI recommendations. These include contexts such as AI chatbot recommendations [25,26], algorithm aversion [27–29], unethical behavior [30], and various consumption scenarios, including hedonic versus utilitarian consumption [31,32].

We particularly adopt the stimulus-organism-response (S-O-R) framework, which explains how external stimuli affect an individual’s internal state, ultimately leading to a behavioral response [33,34]. For instance, previous studies in the marketing domain have investigated various stimuli, including social commerce marketing mixes [35], in-store environments [36], and virtual reality [37]. Based on the initial proposition of the S-O-R framework focused only on emotional responses [33], researchers have extended the types of reactions such as physiology, cognition [38], and long-term memory [34]. In line with this research stream, the current study fills the gaps between existing studies on the S-O-R literature by adding a new dimension of the stimulus of different types of recommendation, such as AI recommendation vs. travel expert recommendation vs. crowdsourced recommendation for the travel destination.

Therefore, this research aims to explore how to integrate emerging technology concepts, such as AI and crowdsourcing, into travel marketing strategies. This study particularly compares traditional destination marketing strategies—where experts serve as the primary source of information. Using two experiments, we offer four main contributions to the literature. First, Study 1 develops insights into and identifies the underlying mechanisms for why people are reluctant to accept AI-generated recommendations [39,40]. Prior AI-related research has mainly focused on the definition and potential usage of AI algorithms in various business fields [41–44]. However, we show that the effect of AI recommendations is limited for travel destination recommendations as consumers have developed a sense of eeriness due to the novelty of the service, thereby decreasing the ads’ effectiveness. Second, we find that the type of consumption motivation (i.e., utilitarian versus hedonic consumption) moderates the effects of recommender type on consumers’ ad evaluation. Using an email advertisement from an online travel agency, Study 2 validates this moderation effect to show that, for the utilitarian motivation condition (i.e., travel for business), the AI recommendation generates a more favorable attitude toward the ad whereas, for the hedonic motivation condition (i.e., travel for a romantic experience), a recommendation from a crowdsourced idea yields the highest level of ad evaluation. Third—and most interestingly—we capture the effect of AI recommendation as being sequentially mediated by decision autonomy and believability only for the utilitarian consumption goal (i.e., business travel). Finally, we establish guidelines for online marketers and advertisers, including immediately actionable advice on how to utilize newly emerging digital technologies (e.g., crowdsourcing and AI) when developing their advertising strategies. Specifically, we detail why consumers evaluate advertisements utilizing different recommenders differently and discuss how to design advertising messages for online travel agencies.

The following sections elaborate on the study’s conceptual backgrounds by integrating the literature streams of advertising strategy for the tourism industry, artificial intelligence, and crowdsourcing under the S – O – R framework [33] and accessibility-diagnosticity framework [45]. Finally, the results, limitations, and suggestions for future research will be discussed.

## Conceptual background

### Marketing and advertising strategy for the tourism industry

In light of the importance of the tourism industry, numerous studies have shed light on the need to build a strategic marketing plan for several market players, including travel agencies [46], hotels [47,48], and even destinations or locations [49,50]. For example, Kim, Franklin [41] investigated the role of price dispersion on hotel preference and found that travelers prefer a hotel option with a wide price dominance dispersion that helps the online travel agency’s pricing strategy. Geng, Le [48] proposed 3D hotel lobby models such as the illuminance of the lamp, the color of the wall, and the decoration style utilizing the eye-tracking method.

Focusing more specifically on the advertising strategy, Stafford [3] pondered whether verbal or visual cues are more effective in hotel advertisements. Subsequently, efforts to figure out effective advertising strategies for the tourism industry have become one of the largest research streams in tourism/service marketing [51,52]. Similar to Stafford’s [3] pioneering work in tourism advertising, early research in this domain has focused predominantly on the effects of design components in tourism advertising. For instance, Decrop [6] found that pictures and texts are the most prevailing elements for ad effectiveness, while Wang and colleagues [5] demonstrated that message appeal types, texts, sizes, and formats are important factors for evaluating group packages of advertisements.

Since the 2010s, researchers have investigated additional antecedents and moderators for ad effectiveness in the tourism context. Pan [53] found that, among several different visual components, familiar objectives using white and blue color schemes generated the most memorable impressions and ultimately increased the recall for New Zealand’s tourism television commercials. In addition, Kemp and colleagues [4] revealed the positive effects of emotional appeals (versus rational appeals) on medical tourism advertising evaluations.

In addition to these insights focusing mainly on the message strategy, other prominent research domains in tourism marketing have included integrating new digital platforms/media to increase marketing effectiveness. Scholars have investigated the effects of virtual reality’s presence in increasing favorable attitudes toward the tourism destination [54], adopting eye-tracking methods to evaluate consumers’ attention in tourism advertisements [55], and analyzing the text from consumers’ reviews [56,57].

Despite these abundant insights, several important unanswered questions remain unanswered, including how best to integrate more recent digital marketing technology/platforms (e.g., crowdsourcing and AI) when developing tourism advertisements.

### AI and crowdsourcing

We adopted the two emerging concepts of AI and crowdsourcing for our main independent variables. AI refers to a broad field of science focusing mainly on the process of learning from data sets and tasks and continuously changing and adopting the optimized outcome from the data [58] in not only the computer science field but also psychology, philosophy, linguistics, and other areas [59]. By integrating “big data,” many business disciplines have rapidly adopted AI; indeed, more than 76% of managers believe that AI technology will change their businesses [58]. Corresponding to this attention from the industry, the Marketing Science Institute [60] addressed the question of “how can one employ AI for better advertising (and customer) engagement?” (p. 7) as a tier 1 priority for the research. In other words, how can advertisers utilize AI to increase ad effectiveness? Several previous studies have documented the potential use of AI algorithms in marketing strategies, such as implementing programmatic advertising [61], automatically targeting high-value customers [41], and proposing financial recommendations [40,42], but a limited number of studies have actually empirically tested AI’s effectiveness in marketing communication strategies.

Another dimension of our independent variable is crowdsourcing, which we defined as the “practice of soliciting travel destination ideas from the crowd” by referencing previous studies rooted in co-creation research [62–65]. Drawing on the basic concepts of co-creation and crowdsourcing detailed in the previous literature, we identified various practices for engaging consumers’ participation in developing marketing strategies. For example, Hazée, Van Vaerenbergh [64] found that engaging consumers in creating their own compensation when they experience an airline service failure buffers their negative response to the service. However, to our knowledge, no previous literature has examined the impact of crowdsourcing on advertising strategy building.

Table 1 summarizes the important and relevant research for the current study.

**Table 1:** Relevant studies.

Author (year)	Independent variable	Moderators/Mediators	Main findings
Castelo (2024)	Algorithm aversion	Perceived corruption	Participants who are born and raised in countries with high levels of perceived corruption are much less averse to algorithmic decision-making
Yu et al. (2024)	Rejection from the Chatbot	Request handling status: failure vs. success)	When consumers receive a rejection of their service request, the evaluation of service is less negative when service is handled by a chatbot agent
Zhu et al. (2023)	AI recommendation	Utilitarian and hedonic consumption	AI recommendation paired with precise numbers and utilitarian consumption generates more positive responses
Kim et al. (2023)	Human vs. non-human agent and unethical behavior	Guilt	Interacting with non-human (vs. human) agents such as AI and robots increases the tendency to engage in unethical consumer behavior due to reduced anticipatory feelings of guilt
Bergner et al. (2023)	Verbal embodiment in conversational AI	Perceived humanness of the interface	Verbal embodiment in conversational AI increases the perceived humanness of the interface for conversational AI, ultimately generating favorable behavioral brand outcomes
Reich et al. (2023)	Algorithm aversion	Perceived learning from mistakes	Trust in a human is higher than in an algorithm for both subjective and objective domains (Study 1), and this is due to the consumer’s perception that the algorithm cannot learn from the mistake (Study 2)
Longoni et al. (2023)	Responses to the AI failure	Algorithmic transference, perception of group homogeneity, discomfort with technology	The more participants report discomfort with new technologies, the more they exhibit transference (study 4). This “algorithmic transference” plays an important role in categorizing AI vs. human agents that ultimately influence an individual’s overgeneralization of the failure of AI
Longoni and Cian (2022)	Artificial intelligence recommendation	Utilitarian and hedonic consumption	The importance of salience of utilitarian attributes determines the preference for AI recommender over human one. In contrast, the importance or salience of hedonic attributes determines resistance to AI recommenders over human ones.

## Hypothesis development

### Stimulus-organism-response (S-O-R) framework

We adopted the Stimulus-Organism-Response (S-O-R) framework, originally proposed by Mehrabian and Russell [33] and modified by Jacoby [34] for the consumer psychology context. This framework suggests that, in a certain environment, individuals’ emotional and cognitive conditions are affected by stimuli, resulting in behavioral outcomes [33,34,66]. The S-O-R framework consists of three components: stimulus, organism, and response. The stimulus is defined as the influence that arouses the individual [34,66,67], while the organism refers to the individual’s cognitive and affective condition that influences the entire process linking stimulus and response [33,66]. Finally, the response is defined as the aggregated consequences that are affected by the stimulus [33,66].

### Accessibility-diagnosticity theory

Based on this basic definition of stimulus, organism, and responses, several studies have investigated various aspects of consumer behavior within the marketing research domain. For instance, the effects of an online store (stimulus) on shopping behavioral outcomes (response) through shoppers’ cognitive and emotional states (organism) have been examined [67]. Similarly, several types of stimuli (social commerce marketing mix, website quality, website reputation, and authentic experience), organisms (value perceptions, pleasure, arousal, dominance, cognitive response, and affective response), and responses (customer loyalty, purchase intention, and attachment) have been scrutinized [35–37,68].

**Fig 1:**
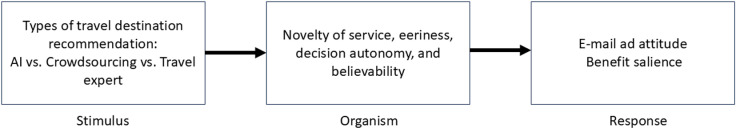
Basic model based on S-O-R framework.

As a theoretical extension, we propose a new type of stimulus (i.e., types of travel destination recommendation), organism (novelty of service, eeriness in Study 1, decision autonomy, and believability for Study 2), and response (email ad attitude and benefit salience) (Fig 1).

We integrate the accessibility diagnosticity theory [45] to extend the type of stimulus and organism. Accessibility-diagnosticity theory focuses on two factors to explain individuals’ information utilization in decision-making processes: accessibility and the diagnosticity of information [45,69–72]. Therefore, we conceptualize the stimulus as both accessible and diagnostic information.

Specifically, the probability that any piece of information will be used as a source for decision making is a function of (1) the accessibility of the information, (2) the accessibility of alternative information, and (3) the diagnosticity and perceived relevance of the information [45,73]. Consumers’ accessibility to the information is associated with the ease of access to consumers’ previous knowledge [74,75], vividness of information [69], consumers’ preexisting memory [70], and proximity between information about brand extension and family brand evaluation [72]. Diagnosticity refers to the degree to “which inferences based on the information alone would be adequate to make a decision” [71], and previous literature found that the perceived importance of information [71] and negativity of information [69] were generally considered to be the more diagnostic information. Following the previous conceptualization of accessibility, we define the level of accessibility as the level of familiarity and ease of access to previous experiences [70,74,75]. As an AI algorithm’s recommendation and the utilization of a humanoid robot in the service industry [39] are considered novel services, consumers will be less familiar with travel destination recommendations from AI. As a result, consumers will be reluctant to generate a positive evaluation toward an email advertisement containing information about the AI-recommended travel destination.

**Fig 2:**
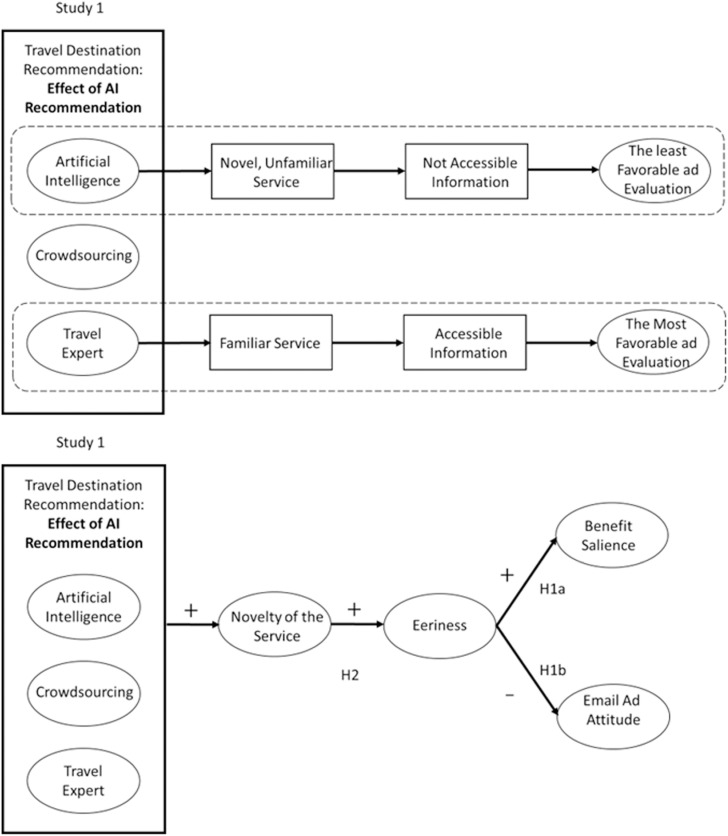
Conceptual and analytic model for Study 1.

In detail, although some studies have revealed the benefits of adopting AI recommendations for both companies and consumers, it is well-documented that consumers might experience algorithm aversion [28,29]. This feeling of aversion may be induced by the lack of trust in the algorithmic recommendation [28] or discomfort with new technologies [27]. Similarly, AI recommendations for travel destinations might be influenced by the novelty of the service, which can evoke a sense of eeriness [39], ultimately reducing the effectiveness of AI recommendations in the consumer decision-making process (Fig 2). Therefore, we posit that:

H1a. Recommendations from AI will produce a higher level of benefit salience toward the company than crowdsourcing recommendations or expert recommendations.

H1b. Recommendations from AI will produce less favorable evaluations of the ad than crowdsourcing recommendations or expert recommendations.

H2. The effect of AI recommendations on (a) ad attitude and (b) benefit salience should be sequentially mediated by the novelty of the service and eeriness.

### Moderating role of consumption goal: Utilitarian versus hedonic

Consumers’ motivations for searching and taking product information are classified into two categories: utilitarian fulfillment and hedonic gratification [76–78]. Utilitarian motivation primarily relates to practical, functional, enabling, and instrumental goals, whereas hedonic motivation is associated with pleasure, fun, enjoyment, and an attractive emotional state [78,79].

**Fig 3:**
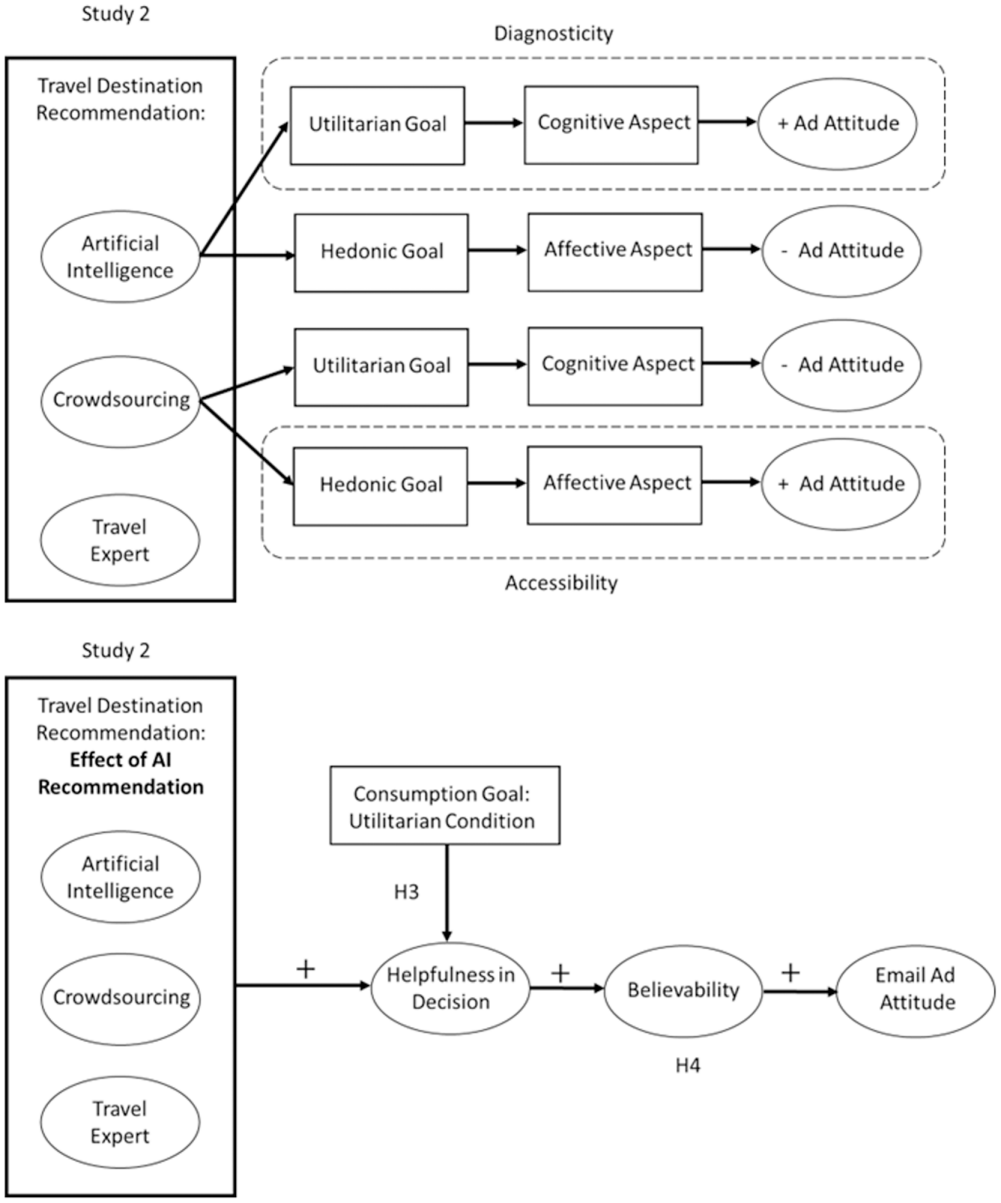
Conceptual and analytic model for Study 2.

Research suggests that utilitarian motivation involves greater use of cognition [80,81] to obtain instrumentally beneficial outcomes, whereas hedonic motivation relates to the emotive aspects of consumers’ experience when they consume products or services to maximize pleasure and minimize pain [80,82]. In Study 2, although participants received a recommendation for the same hotel, the primary goal for the trip differed (i.e., utilitarian goal versus hedonic goal). We manipulated the purpose of the trips as either business trips (utilitarian) or trips for a romantic experience (hedonic). We drew on the accessibility-diagnosticity model, which explained the individuals’ information-seeking and -processing procedures for decision-making. As we elaborated earlier, when participants are exposed to the AI recommendation, they might be reluctant to use this information to foster an attitude toward the ad (Study 1) because this information might not be considered accessible due to the novelty of the service. However, when participants are primed to a more cognition-related condition (i.e., utilitarian condition), they believe that the AI-generated information is more analytic and diagnostic information [45] as they rely on the cognitive aspect of the information that matches the description and definition of AI’s benefits in recommending travel destinations. Therefore, participants in the utilitarian condition feel more decision autonomy from the AI’s travel destination recommendation, and this sense of automated decision is associated with the believability of the recommendation, which ultimately boosts the favorable evaluation toward the email advertisement. Thus, we predict that consumers will have a greater preference for AI recommendations in the context of utilitarian motivation but not hedonic motivation (Fig 3).

H3. There will be an interaction effect of recommender type and consumption goal. The effect of recommender type on ad attitude will be moderated by consumption goal, such that the AI recommendation (versus crowdsourcing and expert recommendations) will generate a more favorable attitude toward the ad in the utilitarian goal condition.

H4. There will be a moderated serial mediation. When a consumer is exposed to the utilitarian goal condition, the effects of the AI recommendation on ad attitude will be sequentially mediated by decision autonomy and believability of the recommendation.

## Study overview

This study was approved by the Institutional Review Board (IRB) of West Virginia State University, with the approval of Dr. Michael Fultz, the IRB Chair. The study was classified under the exempt category, which did not require a written informed consent form. Instead, an online consent form was presented to participants before they were exposed to the experimental stimuli. Only those who agreed to the consent form were allowed to participate in the online experiment.

We conducted two studies to examine our hypotheses. Study 1 investigated the baseline effects of different types of recommenders based on our conceptualization of AI recommendation versus crowdsourcing recommendation versus travel expert. More interestingly, we found the serial mediation effects of the novelty of the service and eeriness perception toward the AI recommendation. By differentiating utilitarian versus hedonic consumption goals, Study 2 investigated the moderated mediation effects through automation in decisions and believability.

## Pilot study

The pilot study aims to select an appropriate image for the travel agency’s email advertisement by ruling out any potential confounding factors. We recruited a total of 306 (*M*_age_: 40.82, *SD* =  13.52, 63.4% female) participants on Amazon’s Mechanical Turk (MTurk), who completed the single factor (3 travel expert images, 5 AI-related images and five crowdsourcing-related images) between-subjects randomized study by indicating the likability of the image (1 =  dislike, 7 =  like), visual appearance of the image (1 =  very bad, 7 =  very good), [78], and the familiarity of the image (7-point semantic differential: unfamiliar/familiar, inexperienced/experienced, not knowledgeable/knowledgeable). The results of a one-way analysis of variance (ANOVA) failed to capture the main effects of liability (*F* (12, 293) =  1.17, *p* = .31) and visual appearance (*F* (12, 293) =  1.67, *p* = .072), but found a significant difference in the familiarity of the image (*F* (12, 291) =  2.91, *p* < .001). Thus, we conducted a follow-up post hoc test using the least significant difference (LSD) method. We excluded the images that exhibited a significantly high level of familiarity and selected one image for each recommender type based on likability (*M*_AI_ =  6.39, *SD* =  2.48 versus *M*_crowdsourcing_ =  6.93, *SD* =  2.02, *M*_expert_ =  6.55, *SD* =  1.82), visual appearance (*M*_AI_ =  6.94, *SD* =  1.92 versus *M*_crowdsourcing_ =  6.71, *SD* =  1.96, *M*_expert_ =  6.83, *SD* =  1.91), and image familiarity (*M*_AI_ =  4.56, *SD* =  1.91 versus *M*_crowdsourcing_ =  4.95, *SD* =  2.11, *M*_expert_ =  5.24, *SD* =  1.99).

## Study 1: Role of AI, crowdsourcing, and experts’ recommendations in deciding travel destination

Study 1 tested our basic prediction that different types of travel recommendations from different sources (i.e., types of recommendation sources) will produce different effects for ad evaluation. In this study, participants were exposed to different types of email advertisements from an online travel company by varying the sources of the recommendation (AI versus crowdsourced idea versus recommendation from the expert) and were asked to evaluate the benefit salience and attitude toward the advertisement.

### Method

#### Participants, design, and procedure.

Initially, 500 participants were recruited; participants who failed to recall the recommendation sources correctly were removed, resulting in a final sample of 450 (*M*_age_: 39.95; SD: 13.38, 60.8% females) on MTurk for the statistical analysis to complete the one-factor (recommender: AI versus crowdsourcing versus expert) between-subjects randomized experiment in exchange for a small monetary compensation. Respondents initially read a brief scenario describing their travel plans to American cities and were asked to choose a hotel. Following this, they were informed that they had subscribed to marketing emails from the fictitious travel agency, Travelsetters.

In this email advertisement, the company introduced the newly adopted travel destination recommendation from the AI, the crowdsourced idea, and the travel expert’s recommendation. All participants were assigned to the conditions of the single factor (AI versus crowdsourcing versus travel expert) between-subjects randomized experiment design. After being exposed to the different descriptions of the recommendation sources, all participants were asked to read an actual recommendation for the same travel destination—namely, The Chanler at Cliff Walk in Newport, Rhode Island, as one of the most romantic hotels in the United States. Finally, they were asked to answer the manipulation check questions for the travel destination, mediators, benefit salience, and attitude toward the ad as dependent variables. After filling out the questionnaire, we also asked for participants’ basic demographic information.

#### Stimuli.

To manipulate our independent variable (recommender type: AI versus crowdsourcing versus travel expert), we created email advertisements containing the announcement of the adoption of a new recommendation system and the actual travel destination recommendation. Three different versions of the email announcement were made by referencing the actual email announcement from the Jetsetter travel agency and the stimulus of a Facebook advertisement utilizing an AI algorithm [40]. In the AI recommender condition, the wording for the email announcement emphasized the usefulness of AI in predicting travelers’ preferences by utilizing the past behaviors of someone who shared similar behavior patterns. For the crowdsourcing condition, the message emphasized the benefit of utilizing other people’s ideas; in the travel expert condition, we indicated that travel experts’ advice could help travelers enjoy future travels and included descriptions of the travel experts by referencing top travel bloggers’ descriptions. For the actual travel destination recommendation advertisement, all participants were exposed to an identical email advertisement for The Chanler at Cliff Walk in Newport, Rhode Island, to minimize the effects of familiarity with the travel destination.

#### Measures.

**Manipulation check:** We measured the effectiveness of the independent variable manipulations (i.e., types of recommender) across the three conditions and evaluated the effects of each condition on the attitude toward the advertising as a dependent variable. We employed two different measurements for the recommender type manipulation. First, participants were directly asked to recall whether they had received the recommendation for their travel plan using a binary response (yes/no) and from whom they received the travel recommendation (AI, crowdsourcing, or travel expert). We then utilized two items, adopted from previous literature, to check the effectiveness of manipulation: (1) “The service provider is like a person” (reverse coded) and (2) “The service provider is machine-like” (Cronbach’s α = .68; [39]).

**Novelty of the service:** One of the most distinct characteristics of AI recommendations is their novelty compared to previous recommendations, including expert recommendations. This perceived novelty affects the adoption of various new technologies [83]. Therefore, we expect that individuals’ different levels of perceived novelty of the service will impact consumers’ decision-making processes. This study measured consumers’ perception of novelty toward the service using two items from a previous study, with modified wording: (1) “I have not been to a service practice like this before” and (2) “This service practice is unusual” (Cronbach’s α = .63; [39]).

**Eeriness:** We also focused on the feeling of eeriness as one of the key mediators to explain the different effects of recommendation sources. Based on humanoid service robots (HSRs) research, the concept of eeriness has been identified as a negative predictor of HSR adoption. Individuals might feel discomfort or creepiness when robots mimic human behavior [39], and this feeling of creepiness can be explained by the concept of the uncanny valley [84]. Therefore, participants indicated their level of eeriness when they received the recommendations from the different sources on three items, using a seven-point Likert-style scale anchored by (1) “This recommendation service is creepy,” (2) “This recommendation service is eerie,” and (3) “This recommendation is unnatural,” also adopted from Mende et al.’s [39] study (Cronbach’s α = .95).

**Benefit salience:** As one of the main dependent variables and mediators, we proposed and measured consumers’ perception of benefit salience for the newly adopted recommender. Originally, corporate social responsibility (CSR) research began by investigating the effects of benefit salience, which refers to the motivation behind firms’ marketing activities, mainly in two ways: for the company’s benefit or for consumers’ benefit [85]. Drawing from this concept of benefit salience, we assume that individuals’ perception of who will benefit more from the adoption of a new AI recommendation system might influence consumers’ decision-making process.

Thus, participants were asked to express their opinion as to who would get more benefit by selecting one of the options: (1) the company (Travelsetters), (2) travelers, or (3) they get the same benefit [86].

**Attitude toward the Ad:** Six items on a 7-point bipolar scale anchored by bad/good, negative/positive, not likable/likable, unfavorable/favorable, not appealing/appealing, and unpleasant/pleasant were adopted from previous literature to measure the attitude toward the email advertisement [87] (Cronbach’s α = .97).

We further investigated the validity and reliability of the measurements by conducting a confirmatory factor analysis for Study 1. The model fit indices showed satisfactory values (Standardized Root Mean Square Residual = .074; Bentler and Bonnet’s Normed Fit Index = .92), as did the discriminant validity indices based on the Heterotrait-Monotrait ratio of correlation (eeriness – ad attitude: .32; eeriness – novelty: .37; novelty of service – ad attitude: .05). Additionally, using the Fornell-Lacker criterion, we confirmed that each construct’s square root of the average variance extracted is greater than the correlation between the construct and any other construct [88–90,91–93].

### Results

#### Manipulation check.

We asked participants to recall directly the source of the recommendation in the email advertisement to which they were exposed. Among the 500 participants initially recruited, 50 participants failed to recall the exact recommendation sources. Next, an ANOVA was performed to examine whether consumers perceived AI, crowdsourcing, and the travel expert differently. As we intended, we found a significant main effect for the recommender type (*F* (2, 447) =  23.30, *p* < .001, partial η^2^ = .09). The mean score for the AI recommendation condition exhibited the highest level of machine likeness (*M*_AI_ =  4.26, *SD* =  1.30), followed by crowdsourcing (*M*_crowdsourcing_ 3.88 =  4.26, *SD* =  1.25), and travel expert (*M*_expert_ =  3.03, *SD* =  1.36). In addition, a subsequent post-hoc analysis using the LSD method reconfirmed the statistical differences among the three conditions.

#### Hypothesis tests.

**Benefit salience:** Regarding Hypothesis 1, we fitted a binary logistic regression model with the dependent variable of benefit salience as a binary choice by collapsing the options into two choices (1 =  company (Travelsetter), and 2 =  travelers/they get the same benefit). We submitted an independent variable as dummy-coded conditions (dummy 0 =  AI, dummy 1 =  crowdsourcing, and dummy 2 =  Travel expert) and found a tendency for participants to indicate that the company received more benefit than users (travelers) in the AI condition (30.9%) compared to the crowdsourcing (22.4%, dummy 1, *b* =  − .47, SE = .26, Wald =  3.17, *p* = .07) and travel expert condition (21.9%, dummy 2, *b* =  − .03, SE = .28, Wald = .01, *p* = .93). These results provided support for our prediction that exposure to the AI recommendation condition would increase the perception of AI being beneficial to the travel agency rather than the travelers. Thus, Hypothesis 1a was supported. Table 2 shows the effects of recommender type on benefit salience.

**Table 2:** Effects of recommender type on benefit salience (Study 1).

	Benefit salience: Who would get more benefit through this recommendation?			b	S.E.	Wald
	Company	Traveler	They can get the same benefit			
**AI Recommendation**	30.9%	36.8%	32.2%	.466	.26	3.17
**Crowdsourcing**	22.4%	39.9%	37.8%	.440	.27	2.74
**Travel Expert**	21.9%	34.2%	43.9%	Reference Group		

Data analyzed by the author from Study 1 dataset.

**Fig 4:**
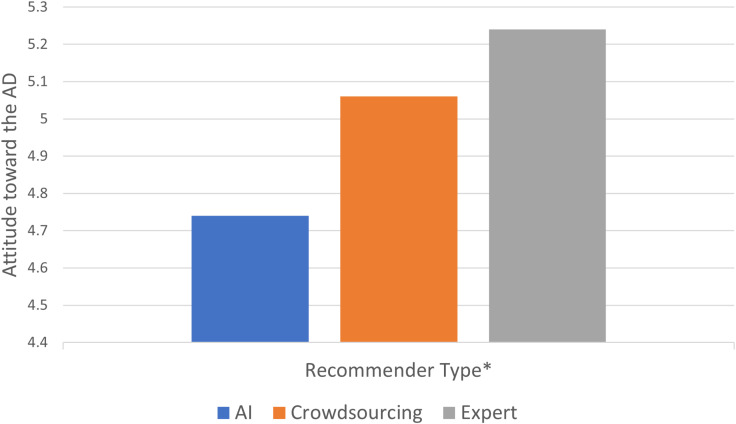
Effects of recommender type on ad attitude (Study 1). * One-way difference significant at *p* < .05.

**Attitude toward the Ad:** We conducted an ANOVA to test Hypothesis 2. We found the significant main effect of recommender type (*F* (2, 442) =  4.61, *p* = .01, partial η^2^ = .02). The mean score for the ad containing the travel expert recommendation generated the most favorite attitude toward the ad (*M*_expert_ =  5.24, *SD* =  1.45), followed by the crowdsourcing recommendation (*M*_crowdsourcing_ =  5.06, *SD* =  1.40); the AI recommendation yielded the least favorable ad attitude (*M*_AI_ =  4.74, *SD* =  1.43). Thus, Hypothesis 1b was supported (Fig 4).

**Mediation analysis:** In terms of our mediation hypothesis (Hypothesis 2), we conducted a series of serial mediation analyses to elucidate the underlying psychological mechanisms driving consumers’ diverse reactions toward the recommender types [94, Model 6]. We entered benefit salience and ad attitude as the dependent variables, recommender type as the independent variable, and novelty of the service and perceived eeriness as mediators. Then, we conducted 10,000 bias-corrected bootstrapping. The results revealed the proposed mediational path (recommendation from AI →  higher level of novelty of service →  greater eeriness →  perceiving the company benefiting more from the AI recommendation) at the 95% confidence interval, especially for the expert recommendation (*b*_crowdsourcing_ = .004, SE = .01, CI _95%_ =  − .03, .04, *b*_expert_ = .06, SE = .02, CI _95%_ = .03, .12). Specifically, (1) types of the recommender using indicator coding and the AI condition as the reference group had a negative relationship with the novelty of the service for the expert recommendation condition (*b*_expert_ =  − .69, *SE* = .16, *t* =  − 4.40, CI _95%_ =  − 1.00,. − .38) as well as the crowdsourcing condition (*b*_crowdsourcing_ =  − .04 *SE* = .16, *t* =  − .27, CI _95%_ =  − .36, .27), (2) novelty of the service had a positive effect on eeriness (*b* = .32, *SE* = .06, *t* =  5.74, CI _95%_ = .21, .43), and (3) eeriness had a negative effect on benefit salience of the traveler (*b* =  − .29., *SE* = .07, CI _95%_ =  − .42, − .17), confirming the statistical significance of the overall logistic regression model (−2LL =  478.14, McFadden’s pseudo *R*^2^ = .05, *p* < .001).

In addition, by adding an indirect path to our analysis model, direct effects from recommender type (independent variable) on benefit salience (dependent variable) were no longer significant, showing full mediation (*b*_crowdsourcing_ = .25, *SE* = .28, CI _95%_ =  − .29, .79, *b*_expert_ = .23, SE = .28, CI _95%_ =  − .31, .77). Neither of the other indirect pathways in this model (e.g., effects of the type of recommendation on benefit salience only through the novelty of the service: CI _95% crowdsourcing_ =  − .04, .05, CI _95% expert_ =  − .07, .21) was significant. We switched the order of the two mediators (i.e., type of the recommender →  eeriness →  novelty of the service), and the indirect effect of the type of the recommender on benefit salience was not significant (*b*_crowdsourcing_ = .01, *SE* = .01, CI _95%_ =  − .02, .04, *b*_expert_ = .01, *SE* = .01, CI _95%_ =  − .02, .04).

**Fig 5:**
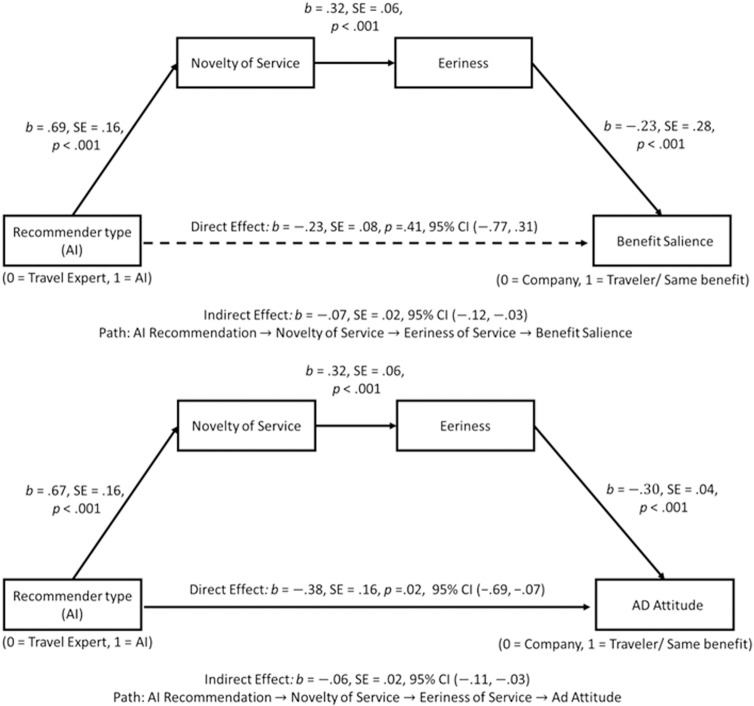
Serial mediation results (Study 1).

We conducted the same serial mediation analysis and found a similar pattern in the effects of recommender type on the attitude toward the ad through the novelty of the service and eeriness. Specifically, the type of recommender was a negative predictor of the novelty of the service in only the travel expert condition (*b*_expert_ =  − .67, *SE* = .16, *t* =  − 4.22, CI _95%_ =  − .98, − .36, *b*_crowdsourcing_ =  − .01 *SE* = .16, *t* =  − .07, CI _95%_ =  − .33, .31). This novelty of the service was a positive predictor of the level of eeriness (*b* = .32 *SE* = .06, *t* =  5.63, CI _95%_ = .21, .43) that was ultimately negatively associated with attitude toward the ad (*b* =  − .30 *SE* = .04, *t* =  − 7.50, CI _95%_ =  − .37,. − .22), by showing a significant overall model fit (*R*^2^ = .13, *F* (4.439) =  16.66, *p* < .001). We reversed the order of the mediators, and the indirect effects for both conditions were not significant (CI _95% crowdsourcing_ =  − 0.03, 0.0003, CI _95% expert_ =  − .03, .0003). In addition, when we controlled the indirect effects through the mediators, the direct effect of the expert on ad attitude was still significant (*b* = .38 *SE* =  0.16, *t* =  2.39, CI _95%_ =  .07, .69), indica*t*ing a partial mediation result (supporting Hypothesis 2, Fig 5).

### Discussion

Study 1 provided the baseline evidence for our main prediction: adopting an AI recommendation system sometimes fails to lead tourism consumers to generate a positive attitude toward the ad and make them believe that the newly adopted recommendation system is beneficial for them. Indeed, consumers exhibited the most positive attitude toward the email advertisement when the expert recommended the travel destination, and they believed that the travel agency genuinely helped the customers and then benefitted more by adopting the recommendation from the travel expert. Through sequential mediation analysis, we delved deeper into the underlying mechanisms to understand why people hesitated to follow the AI-generated recommendation. Compared to the crowdsourced recommendation and the expert’s recommendation, the newly adopted AI algorithm was considered a novel and unusual service, resulting in feelings of eeriness when it recommended a travel destination for the consumers. This situation ultimately led to benefit salience for the company and the least favorable attitude toward email advertising.

Although we found the baseline effects of different types of recommendation sources (i.e., recommendation from AI versus crowdsourcing versus travel experts), consumers’ travel information search behavior might be influenced by the goal of their travel. Thus, having recognized the moderating role of consumption type (utilitarian versus hedonic), we next investigated the role of consumption motivation on attitude toward email advertisement.

## Study 2: Moderating role of consumption goal (hedonic versus utilitarian)

Study 2 sought to show an important boundary condition for our proposed independent variables by testing the moderating role of consumption type. As previously discussed, consumers’ information search behavior can be focused on either utilitarian or hedonic motivations [76–78]. By integrating two research streams of accessibility-diagnosticity [45] and consumption goal [76], we proposed that if consumers’ information-processing motivation was framed as utilitarian (i.e., business trip), the AI recommendation would be considered more diagnostic information, thereby yielding a more favorable ad evaluation compared to the hedonic-framed condition (i.e., romantic trip).

### Method

#### Participants, design, and procedure.

We recruited a total of 600 participants through MTurk but excluded 49 individuals who failed to recall the exact recommender (similar to Study 1). Thus, we utilized a total of 551 participants for our statistical analysis (*M*_age_: 38.28, *SD* =  13.08, 56.8% female). Study 2 employed a 3 (recommender type: AI versus crowdsourcing versus travel expert) ×  2 (consumption type: utilitarian versus hedonic) between-subjects design. For the first independent variable of recommender type, we utilized the same stimuli as in Study 1. For the second independent variable, we varied the consumption motivation by giving different recommendations based on the conditions. Specifically, after being exposed to the email advertisement about the recent adoption of a travel destination recommendation system utilizing AI (versus crowdsourcing versus travel expert) from the same fictitious travel agency (i.e., Travelsetters), participants were asked to read a brief scenario that they needed to travel to New York City for a business trip (versus romantic experience). We purposely selected New York City for several reasons. First, New York City has been ranked second in the list of the best cities for business travel [95], and previous studies manipulating the consumption type have frequently utilized New York City as a travel destination to test different consumption motivations [e.g., 96]. Thus, we selected Gramercy Park Hotel in New York City as this hotel is known as one of the best hotels for both business trips [97] and romantic experiences [98].

After participants were exposed to one of the first independent variable conditions by receiving an email advertisement regarding the newly adopted travel destination recommendation system, they were asked to read another email advertisement for the actual hotel recommendation based on the assigned condition (utilitarian versus hedonic). Two different versions of a single page, color email advertisements were designed to manipulate the consumption type. For the utilitarian condition, we first asked participants to imagine they were planning to travel to New York City for a business trip.

#### Measures.

For Study 2, we utilized the same manipulation check question for the recommender type [39] and the dependent variable of attitude toward the ad [87] (Cronbach’s α = .95).

**Manipulation check:** We also used two questions for the consumption type manipulation check. First, we directly asked participants to recall the purpose of the trip with a binary choice (1 =  business, 2 =  romantic experience). We then asked them to indicate whether they perceived the recommendation for their travel as utilitarian or hedonic (1 =  primary utilitarian – 7 =  primary hedonic) by modifying the wording from the previous literature [96].

**Mediators:** Study 2 employed two mediators. Decision autonomy was assessed using a single item anchored by “The message tried to make a decision for me” (1 =  strongly disagree/ 7 =  strongly agree). The believability of recommendation was evaluated using a single item (ranging from 1 =  not at all to 7 =  completely) [99].

**Trustworthiness of human recommender:** As the previous literature found, the trustworthiness of humans and algorithms can vary across several recommended behaviors [40], we measured the level of trustworthiness of the human recommender as a control variable for Study 2. We asked the participants to express their level of trust when they received the recommendation from the human by moving the slider from 0 to 100 [40].

### Results

#### Manipulation check.

We first excluded all the respondents who failed to recall the recommender (AI versus crowdsourcing versus travel expert) and the consumption type (utilitarian versus hedonic). A total of 49 participants failed to recall either the recommender or the consumption type; thus, we excluded these participants from the statistical analysis. We submitted a 3 (recommender type: AI versus crowdsourcing versus expert) ×  2 (consumption type: utilitarian versus hedonic) ANOVA with machine-likeness as a dependent variable. We found the main effect of recommender type (*F* (2, 545) =  13.67, *p* < .001, partial η^2^ = .05). The AI condition generated the highest level of machine likeness (*M*_AI_ =  4.70, *SD* =  1.45), followed by the crowdsourcing (*M*_crowdsourcing_ =  4.24, *SD* =  1.43) and expert conditions (*M*_expert_ =  3.84, *SD* =  1.77), indicating that our manipulation of the recommender type was successful. A follow-up post-hoc analysis utilizing LSD indicated that the three conditions generated statistically different evaluations of machine-likeness. None of the main effects of consumption type (*F* (1, 545) = .14, *p* = .71, partial η^2^ = .000) or the interaction between recommender type and consumption type were significant (*F* (2, 545) = .23, *p* = .79, partial η^2^ = .001).

A two-way ANOVA on the utilitarian–hedonic feeling only showed a significant main effect of consumption type (*F* (1, 545) =  337.68, *p* < .001, partial η^2^ = .38), with the hedonic consumption condition generating a higher level of hedonic feeling (*M*_hedonic_ =  5.95, *SD* =  1.22 versus *M*_utilitarian_ =  3.38, *SD* =  1,99). In addition to the main effect of consumption type, the main effect of recommender type (*F* (2, 544) = .74, *p* =  .48, partial η^2^ =  .003) and the interaction between recommender type and consumption type (*F* (2, 544) =  .98, *p* = .38, partial η^2^ =  .004) were not significant.

#### Hypothesis testing.

**Fig 6:**
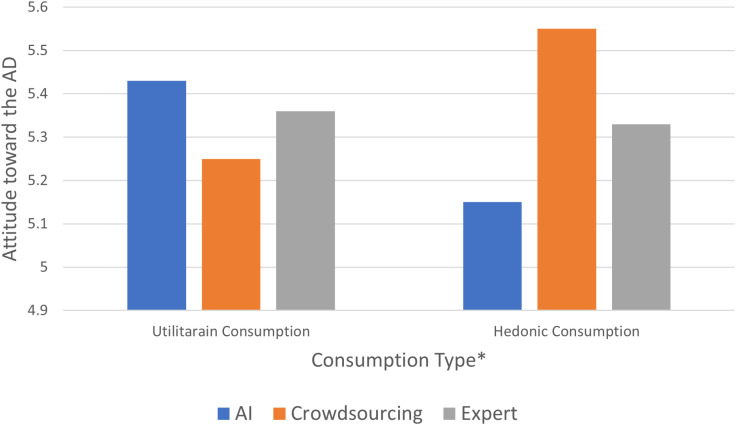
Effects of recommender type on ad attitude (Study 2). * Two-way difference significant at *p* < .05.

**Ad attitude:** A two-way ANOVA on ad attitude with the trust level of the human recommender as a covariate yielded the predicted interaction (F (2, 536) =  2.93), *p* = .05, partial η^2^ = .01) by showing the moderating effect of consumption type. Specifically, for the utilitarian consumption condition, the AI recommendation generated the most favorable attitude toward the ad, followed by the expert recommendation and the crowdsourcing condition, which yielded the least favorable attitude toward the ad (*M*_AI_ =  5.43, *SD* =  1.21 versus *M*_crowdsourcing_ =  5.25, *SD* =  1.24, versus *M*_expert_ =  5.36, *SD* =  1.24). Meanwhile, we found the opposite pattern for the hedonic consumption, with the highest level of ad favorability for crowdsourcing (*M*_crowdsourcing_ =  5.55, *SD* =  1.19), followed by expert (*M*_expert_ =  5.25, *SD* =  1.25) and AI (*M*_AI_ =  5.15, *SD* =  1.23). Thus, Hypothesis 3 was supported (Fig 6).

**Moderated serial mediation:** Based on our conceptualization, decision autonomy and believability would sequentially mediate the effects of recommender type on consumers’ email ad evaluation only for the utilitarian consumption condition. To further examine the underlying mechanism for this mechanism, we conducted a moderated sequential mediation analysis with recommender type as an independent variable, decision autonomy and believability of the recommendation as mediators, and the ad attitude as a dependent variable. We conducted three sets of serial mediations [94; model 6, 10,000 bootstrapping samples] to test our focal pathway: AI- (versus crowdsourcing versus expert) recommendation →  decision autonomy →  believability →  ad attitude by following the previous literature’s guidelines [100–102]. Specifically, we first tested the combined condition’s serial mediation path, and none of the direct (*b*_crowdsourcing_ = .01, *SE* = .09, *t* = .06, *p* = .96, CI _95%_ =  − .17, .18; *b*_expert_ =  − .12, *SE* = .09, *t* =  − 1.28, *p* = .20, CI _95%_ =  − .30, .06) or indirect effects (*b*_crowdsourcing_ =  − .02, *SE* = .01, CI _95%_ =  − .05, .005; *b*_expert_ =  − .013, *SE* = .01, CI _95%_ =  − .04, .01) on ad attitude were significant. However, a significant indirect effect emerged through the decision autonomy and believability on ad attitude for utilitarian consumption condition (*b*_crowdsourcing_ =  − .06, *SE* = .03, CI _95%_ =  − .13, − .004; *b*_expert_ =  − .05, *SE* = .03, CI _95%_ =  − .13, − .003). Other direct paths and mediation paths using a single mediator were not significant, confirming that the sequential mediation path was the only significant path (overall model fit: *R*^2^ = .51, *F* (4, 248) =  66.10, *p* < .001). In the hedonic condition, we failed to capture any significant direct or indirect effects through the mediators, suggesting that the AI recommendation generated a favorable evaluation toward the email advertisement only through the decision autonomy, which led to a higher level of believability that ultimately increased the favorable attitude toward the email advertisement when consumers search for travel destination information for utilitarian motivation. To rule out an alternative explanation, we switched the order of the mediator (believability of recommender →  decision autonomy) and executed the same moderated serial analysis. None of the direct effects or indirect effects were significant (supporting Hypothesis 4).

**Additional analysis:** To provide further support for our findings, we also tested several alternative explanations for our effects: attractiveness of the recommendation, credibility of the recommendation, and perceived characteristics of the recommender (warmth, emotional appeal, intelligence). We analyzed each in a separate model using attitude toward advertising as a dependent variable and our independent variables (recommender type and consumption type) as predictors. Across these six additional analyses, no interaction effects were significant (*F* <  2.03, NS), confirming that none of these constructs could account for our observed effects.

### Discussion

By showing that the effects of recommender type are moderated by consumption goal and mediated by the decision autonomy and believability, Study 2 offered evidence for the underlying mechanism driving this effect. In addition, when we did not divide the consumption type (Study 1), the overall effects of the recommender type were the most powerful when participants were exposed to the travel expert’s recommendation. However, we found that this result could be explained by the fact that the effects of the AI recommendation and crowdsourcing canceled each other out when we aggregated the sample by providing evidence of the moderating effect of the consumption type.

## General discussion

Prior research has provided abundant evidence on how different information sources influence people’s decision-making processes. Based on the accessibility and diagnosticity framework [45], previous research has shown that vividness of information [69], the proximity between brand extension information and family brand evaluation [72], and consumers’ prior attitude or preexisting memory [70] can be used as accessible information for consumers’ decision-making process. By adding knowledge to this growing body of literature, the current study has proposed that novelty to the service can be another source for providing accessible information for individuals’ decision-making process when using recommendations from the novel AI algorithm. Study 1 showed that the advertisement containing the recommendation from the travel expert generated the most favorable evaluation for the email advertisement, whereas the AI algorithm recommendation yielded the least favorable ad evaluation. We replicated this result by changing the dependent variable as a benefit salience; thus, consumers believed that implementation of the AI recommendation was for the company’s benefit and less beneficial for the consumer’s welfare. We explained these different effects of recommender type as a function of the novelty of service and the eeriness perception toward the AI recommendation. This study also demonstrated the moderating role of the consumption goal in showing that the AI recommendation (versus crowdsourcing versus expert) in the travel destination ad under the utilitarian goal condition yielded the most favorable ad evaluation, but under the hedonic goal condition, the ad implementing a crowdsourced travel destination recommendation fostered the most favorable ad evaluation (Study 2). We proposed the underlying psychological mechanism of the mediating role of decision autonomy and believability. These results offer significant theoretical and managerial implications.

### Theoretical implications

Drawing on the overarching theoretical framework of accessibility-diagnosticity [45], we explored a novel type of accessible and diagnostic information in the tourism advertising context: recommendation from a familiar expert (accessible information) and diagnostic information (recommendation from AI). Our work also extends existing research on how individuals evaluate the information based on the consumption goal type. Previous works from the consumption goal literature have revealed the different effects of hedonic versus utilitarian consumption, focusing mainly on the satisfaction of consumption and other emotional rewards of consumption. Our findings contribute to the existing knowledge base by incorporating several mediators to explain this moderation role of the consumption goal.

Second, in exploring how consumers perceive the information from the newly developed digital platform of crowdsourcing and AI, our work offers a new perspective to the literature on digital advertising and marketing. Prior research primarily demonstrated the positive effects of digital technology on communicating with consumers [61,103]. However, contrary to the laypeople’s belief that utilizing AI or crowdsourcing is always beneficial to the company or organization, Terblanche, Molyn [104] found that there is a specific condition to maximize the effectiveness of an AI coach in training people due to the lack of empathy and emotional intelligence. Similarly, our studies demonstrated that we should focus on the boundary conditions to maximize the effects of AI-generated and crowdsourced recommendations [104,105].

More broadly, our work extends the existing understanding of the effects of a communicator rooted in the source credibility literature [106]. Previous work has largely focused on the effects of different source characteristics, such as source attractiveness [107] and expertise [108]. In demonstrating the underlying mechanism of why AI and crowdsourcing generated different levels of ad effectiveness with several mediators (e.g., the novelty of service, the feeling of eeriness, helpfulness in decision, and believability), we provide a novel insight into digital marketing literature.

Finally, in exploring the joint effects of different recommendation source types and consumption goal types in the tourism advertising/marketing context, we integrated literature streams from digital marketing and tourism marketing. Previous work in the tourism advertising literature has mainly focused on the message’s appeal; to the best of our knowledge, the current work is the first to investigate whether people would utilize different recommendation sources when their consumption motivation varied.

### Practical, managerial implications, and strategic defaults

Our research provides several strategic implications for marketers and advertisers. First, as more companies have adopted AI technology for their services (e.g., Aloft Hotel’s virtual chatbot, Zillow.com’s home/apartment recommendation), online travel agencies such as Expedia.com have also utilized the AI recommendation system to suggest hotels when consumers purchase airline tickets. Our findings offer guidelines for how marketers and advertisers can select an appropriate match for travel destination recommendations. First, marketers should avoid utilizing an AI recommendation without considering their travel product type. Implementing an AI recommendation for a travel destination is only beneficial for the utilitarian trip (i.e., business trip), whereas a trip for hedonic motivation (i.e., for a romantic experience) benefits from either a travel expert recommendation (Study 1) or crowdsourced recommendation (Study 2).

In addition, advertisers should recognize the fundamental mechanism for the different effects of recommender type by considering our proposed mediators. Considering the full mediation results from Study 1, when advertisers make an ad implementing the AI recommendation, it is important to implement the message component in order to reduce the feeling of extreme novelty, which turns to feelings of eeriness. Thus, directly implementing a familiar humanoid face [39], anthropomorphized symbol [109], or name for the AI (e.g., Botlr for the Aloft Hotels chatbot) for the advertising design components might be one great solution to increase familiarity.

When firms design the advertising message for utilitarian travel, our research suggests that advertisers should consider the perceived benefit of the AI recommendation system by mentioning how the recommendation system helps customers make decisions (decision autonomy in Study 2). This feeling of automated decision can then be associated with the believability of the recommendation system, which ultimately increases the ad effectiveness. Therefore, we recommend adding a message to advertisers to emphasize the benefit that consumers might enjoy when they adopt the recommendation from the AI algorithm. Using our guidelines and detailed suggestions for advertisers who want to utilize the AI recommendation system, online travel agencies and advertisers can work together strategically to optimize the performance of email advertisements by utilizing different types of recommenders that match consumers’ travel goals.

### Boundaries and extensions

Despite the robustness of our findings and the converging psychological evidence proposed, our research has limitations, offering several opportunities for future research. We focus our discussion on four directions.

First, this study provided evidence for the travel industry by focusing on the experiential dimension of consumption [110,111]. In addition to the utilitarian versus hedonic motivation differentiation, the same trip to New York City might differ in terms of the experiential versus material consumption differentiation [96]. For example, consumers might want to travel to New York City mainly for shopping purposes (material consumption), whereas other consumers might want to enjoy musical performances (experiential consumption). Considering the different effects of recommender type (AI versus crowdsourcing versus expert), consumption type might moderate the effects of AI recommendations.

Given the increasing number of AI recommendations from various digital platforms (e.g., Apple’s and Spotify’s music recommendations and Zillow’s real estate recommendations), consumers often face the dilemma of choosing which recommendation to follow for optimal results. For instance, when individuals need to decide on renting an apartment for the coming year, they may choose to rely on the recommendation from Zillow’s AI algorithm or seek advice from a realtor. Our framework can be applied to studies of the different effects of recommender type by integrating the construct of involvement (music recommendation from Spotify—low involvement versus renting an apartment using Zillow.com—high involvement). In addition, the current study did not consider the effects of the COVID-19 pandemic on travelers’ decision-making processes [112,113]. Therefore, a follow-up study should investigate the effects of different types of information sources (e.g., AI-generated vs. expert-recommended), channels (e.g., email marketing vs. social media posting), and their interactions by comparing individuals’ behavior before and after COVID-19. Additionally, recent advances in machine learning techniques, particularly in computer vision [15] and natural language processing [114–118], should be implemented for analyzing large social big data sets to capture consumer responses to AI recommendations.

Finally, future research could extend our study’s findings by integrating individual differences, such as self-construal [119], and studying whether recommender type influences consumers’ decision-making process differently based on the individuals’ self-view.
